# Supplementary material for: Application of relay puncture technique in treating patients with complicated lower extremity arterial diseases
Source: PeerJ. 2019 Feb 13;7:e6345. doi: 10.7717/peerj.6345 (PMC6377597; doi:10.7717/peerj.6345)
Supplement: Supplemental Information 1 [file peerj-07-6345-s001.doc]

AA. Raw data of patients’ history in the present study

| ID | Gender | Age | Date | Symptom | Hypertension | Diabetes | CD | Smoke |
| --- | --- | --- | --- | --- | --- | --- | --- | --- |
| 323192 | M | 71 | 2014.12.16 | Extensive necrosis | Y | Y | Y | Y |
| 341087 | F | 81 | 2015.2.3 | Rest pain | Y | N | Y | Y |
| 296311 | M | 80 | 2015.5.26 | Rest pain | Y | Y | N | N |
| 368721 | M | 61 | 2015.9.7 | Local ulcer | Y | N | N | Y |
| 401353 | M | 70 | 2015.12.31 | Rest pain | Y | Y | N | Y |
| 402120 | F | 64 | 2016.1.10 | Extensive necrosis | Y | Y | N | Y |
| 402317 | M | 57 | 2016.1.22 | Serious claudication | Y | Y | Y | N |
| 404174 | M | 66 | 2016.2.10 | Rest pain | Y | Y | Y | Y |
| 405293 | M | 75 | 2016.3.5 | Local ulcer | Y | N | Y | Y |
| 408318 | M | 72 | 2016.5.4 | Local ulcer | Y | N | Y | Y |
| 349674 | M | 77 | 2016.6.22 | Rest pain | Y | Y | N | Y |
| 410912 | F | 81 | 2016.7.1 | Extensive necrosis | N | N | Y | N |
| 412302 | F | 55 | 2016.10.29 | Rest pain | Y | Y | Y | Y |
| 415815 | M | 48 | 2016.11.30 | Rest pain | Y | Y | N | Y |
| 415901 | M | 56 | 2016.12.5 | Local ulcer | Y | Y | N | Y |
| 417612 | M | 70 | 2017.1.19 | Extensive necrosis | Y | N | Y | N |
| 351822 | F | 79 | 2017.2.1 | Local ulcer | N | Y | N | N |
| 256013 | M | 71 | 2017.2.12 | Rest pain | Y | N | Y | Y |
| 418011 | M | 59 | 2017.3.4 | Extensive necrosis | Y | Y | Y | Y |
| 420876 | M | 63 | 2017.4.22 | Serious claudication | Y | N | N | Y |
| 422130 | M | 69 | 2017.6.5 | Extensive ulcer | Y | Y | Y | Y |

CD: coronary disease

BB. Raw data of patient symptoms

| **Patient ID** | **Before treatment** | | | | | **Immediately after treatment** | | | | **3 months after treatment** | | | | **6 months after treatment** | | | | **12 months after treatment** | | | |
| --- | --- | --- | --- | --- | --- | --- | --- | --- | --- | --- | --- | --- | --- | --- | --- | --- | --- | --- | --- | --- | --- |
|  | **RCC** | | | **ABI** | | **RCC** | | **ABI** | | **RCC** | | **ABI** | | **RCC** | | **ABI** | | **RCC** | | **ABI** | |
| 1 | 6 | | 0 | | | 0 | | 0.55 | | 0 | | 0.63 | | 1 | | 0.58 | | 4 | | | 0.42 |
| 2 | 4 | | 0.51 | | | 0 | | 0.81 | | 0 | | 0.79 | | 0 | | 0.83 | | 0 | | | 0.65 |
| 3 | 4 | | 0.48 | | | 0 | | 0.93 | | 0 | | 0.90 | | 0 | | 0.88 | | 0 | | | 0.92 |
| 4 | 5 | | 0.22 | | | 0 | | 0.68 | | 0 | | 0.71 | | 0 | | 0.68 | | 0 | | | 0.77 |
| 5 | 4 | | 0.36 | | | 0 | | 0.99 | | 0 | | 1.03 | | 0 | | 0.98 | | 0 | | | 0.95 |
| 6 | 6 | | 0 | | | 1 | | 0.70 | | 1 | | 0.82 | | 1 | | 0.79 | | 4 | | | 0.58 |
| 7 | 3 | | 0.60 | | | 0 | | 1.08 | | 0 | | 1.05 | | 0 | | 1.02 | | 0 | | | 0.98 |
| 8 | 4 | | 0.41 | | | 0 | | 0.93 | | 0 | | 0.93 | | 0 | | 0.88 | | 0 | | | 0.80 |
| 9 | 5 | | 0.45 | | | 0 | | 1.12 | | 1 | | 1.05 | | 0 | | 1.02 | | 0 | | | 0.95 |
| 10 | 5 | | 0.36 | | | 0 | | 0.81 | | 0 | | 0.88 | | 0 | | 0.90 | | 0 | | | 0.89 |
| 11 | 4 | | 0.72 | | | 0 | | 1.10 | | 0 | | 1.00 | | 0 | | 0.98 | | 0 | | | 0.96 |
| 12 | 6 | | 0 | | | 1 | | 0.69 | | 1 | | 0.73 | | 1 | | 0.63 | | 5 | | | 0.36 |
| 13 | 4 | | 0.58 | | | 0 | | 1.03 | | 0 | | 1.05 | | 0 | | 0.95 | | 0 | | | 0.90 |
| 14 | 4 | | 0.35 | | | 0 | | 0.95 | | 0 | | 0.99 | | 0 | | 0.86 | | 0 | | | 0.79 |
| 15 | 5 | | 0.33 | | | 0 | | 0.88 | | 0 | | 0.80 | | 0 | | 0.79 | | 0 | | | 0.66 |
| 16 | 6 | | 0.22 | | | 0 | | 0.93 | | 0 | | 0.99 | | 0 | | 0.85 | | 4 | | | 0.45 |
| 17 | 5 | | 0.17 | | | 0 | | 0.99 | | 0 | | 0.97 | | 0 | | 0.91 | | 1 | | | 0.86 |
| 18 | 4 | | 0.32 | | | 0 | | 1.02 | | 0 | | 1.00 | | 0 | | 0.89 | | 0 | | | 0.92 |
| 19 | 6 | | 0 | | | 1 | | 0.76 | | 1 | | 0.77 | | 1 | | 0.65 | | 3 | | | 0.53 |
| 20 | 3 | | 0.45 | | | 0 | | 1.10 | | 0 | | 1.01 | | 0 | | 0.93 | | 0 | | | 0.93 |
| 21 | 6 | | 0.22 | | | 0 | | 0.93 | | 0 | | 0.99 | | 0 | | 0.85 | | 4 | | | 0.45 |
| Median/Mean | 5 | 0.33±0.18 | | | 0 | | 0.90±0.16* | | 0 | | 0.91±0.13* | | 0 | | 0.85±0.16* | | 0 | | 0.75±0.21* | | |

RCC, Rutherford Clinical Classification. Least significant difference *t*-test, **P*<0.05 (both compared with ABI/RCC before treatment). The median value is for RCC, and the mean value is for ABI, which are presented as mean ± standard deviation.

CC. Raw data of patient symptoms

| **Patient ID** | **Before treatment** | **3 months after treatment** | **6 months after treatment** | **12 months after treatment** |
| --- | --- | --- | --- | --- |
| 1 | Extensive necrosis | Minor amputations have been completed and the wound healed well | Mild claudication | Severe claudication |
| 2 | Rest pain | No symptoms recurred | No symptoms recurred | No symptoms recurred |
| 3 | Rest pain | No symptoms recurred | No symptoms recurred | No symptoms recurred |
| 4 | Local ulcer | wound healed well | No symptoms recurred | No symptoms recurred |
| 5 | Rest pain | No symptoms recurred | No symptoms recurred | No symptoms recurred |
| 6 | Extensive necrosis | Minor amputations have been completed and the wound healed well; Mild claudication | Mild claudication | Severe claudication |
| 7 | Serious claudication | No symptoms recurred | No symptoms recurred | No symptoms recurred |
| 8 | Rest pain | No symptoms recurred | No symptoms recurred | No symptoms recurred |
| 9 | Local ulcer | The wound healed well with minor claudication | No symptoms recurred | No symptoms recurred |
| 10 | Local ulcer | The wound healed well | No symptoms recurred | No symptoms recurred |
| 11 | Rest pain | No symptoms recurred | No symptoms recurred | No symptoms recurred |
| 12 | Extensive necrosis | Minor amputations have been completed and the wound healed well | Minor claudication | Rest pain recurred |
| 13 | Rest pain | No symptoms recurred | No symptoms recurred | No symptoms recurred |
| 14 | Rest pain | No symptoms recurred | No symptoms recurred | No symptoms recurred |
| 15 | Local ulcer | No symptoms recurred | No symptoms recurred | No symptoms recurred |
| 16 | Extensive necrosis | Minor amputations have been completed and the wound healed well | No symptoms recurred | Severe claudication |
| 17 | Local ulcer | The wound healed well | No symptoms recurred | No symptoms recurred |
| 18 | Rest pain | No symptoms recurred | No symptoms recurred | No symptoms recurred |
| 19 | Extensive necrosis | Minor amputations have been completed and the wound healed well | Minor claudication | Moderate claudication |
| 20 | Serious claudication | No symptoms recurred | No symptoms recurred | No symptoms recurred |
| 21 | Extensive ulcer | Minor amputations have been completed and the wound healed well | No symptoms recurred | Severe claudication |
